# The structure and assembly of rhizobacterial communities are influenced by poplar genotype

**DOI:** 10.3389/fmicb.2022.1052567

**Published:** 2022-11-29

**Authors:** Qi Liang Zhu, Kun Yan, Nian Zhao Wang, Shu Qi Ma, De Shan Lu, Xiao Hua Su, Zheng Sai Yuan, Yu Feng Dong, Yan Ping Wang, Chang Jun Ding

**Affiliations:** ^1^Taishan Forest Ecosystem Research Station of State Forestry Administration, College of Forestry, Shandong Agricultural University, Tai’an, China; ^2^State Key Laboratory of Tree Genetics and Breeding, Research Institute of Forestry, Chinese Academy of Forestry, Beijing, China; ^3^Key Laboratory of Tree Breeding and Cultivation of State Forestry Administration, Research Institute of Forestry, Chinese Academy of Forestry, Beijing, China; ^4^Shandong Academy of Forest, Jinan, China

**Keywords:** poplar genotype, rhizobacterial community, community assembly, co-occurrence network, plant-microbe relationship

## Abstract

The interaction between plants and microbes dominates plant growth and fitness in specific environments. The study of the relationship between plant genotypes and rhizobacterial community structure would provide a deep insight into the recruitment strategies of plants toward soil bacteria. In this study, three genotypes of 18-year-old mature poplar (H1, H2, and H3) derived from four different parents were selected from a germplasm nursery of *Populus deltoides*. Rhizosphere soil carbon, nitrogen, and phosphorus properties as well as the 16S rDNA sequences of rhizobacterial communities were analyzed to determine the relationship between poplar genotypes and rhizobacterial communities assembly. The results showed there were significant differences in the diversity (Chao1, ACE index, and Shannon index) of rhizobacterial communities between H1 and H2, as well as between H2 and H3, but no difference between H1 and H3. Principal component analysis also revealed a similar structure of rhizobacterial communities between H1 and H3, whereas the rhizobacterial communities of H2 demonstrated significant differences from H1 and H3. Linear discriminant effect size analysis indicated that there were 11 and 14 different biomarkers in the H1 and H3 genotype, respectively, but 42 in the H2 genotype. Co-occurrence network analysis indicated that the rhizobacterial communities of H2 had a distinct network structure compared to those of the other two genotypes, whereas H1 and H3 had a similar pattern of co-occurrence network. Threshold indicator taxa analysis revealed that 63 genera responded significantly to NO_3_^–^-N content and 58 genera to NH_4_^+^-N/NO_3_^–^-N ratio. Moreover, the stochastic assembly process was found to be decreased with increasing NO_3_^–^-N content and fluctuated with increasing NH_4_^+^-N/NO_3_^–^-N ratio. All results indicated that the structure of poplar rhizobacterial communities were influenced by host genotypes, and available nitrogen might play a dominant role in the assembly of rhizobacterial communities. This study would promote the future selection and utilization of rhizobacteria in poplar breeding.

## Introduction

Rhizosphere microorganisms play key roles in plant growth and fitness (Ren et al., 2020). Studies on the relationships between plants and microbes are showing profound impacts on modern agriculture. Researchers are trying to employ diverse microbes to improve plant growth and tolerance to stress. The relationship between soil habitat and microbial community has got a lot of attention (Williams and de Vries, 2020), while the interaction between soil microbiota and plant genotype is often ignored (Hemmerle et al., 2022; Qi et al., 2022). The genetic conservation makes the morphological and physiological traits of progenies biologically equivalent to their parents (Zanewich et al., 2018). However, hybridization between parents with different genotypes could create some new distinct phenotypic traits (Shen et al., 2021), so that hybrid progenies usually demonstrate more advantages of phenotype (Zanewich et al., 2018). The genetic distances between parent lines seem to be responsible for the progeny phenotype in out-crossing plants. Theoretically, rhizosphere microbial communities of plants are related to plant breeding practice. For example, a recent study showed that self-cross of rice (*Oryza* sp.) decreased the differences in rhizosphere bacterial and fungal communities between parental lines and progeny generations (Chang et al., 2021). So, it is necessary to combine traditional plant cross-breeding with soil microbiomes (Nerva et al., 2022). The genotypic differences of host plants may also play more significant roles in the recruitment and assembly of the rhizosphere microbiome (Cregger et al., 2018; Morella et al., 2019; Veach et al., 2019), thereby determining the plant growth and fitness (Brachi et al., 2022). Although the impact of host genotype on the assembly of rhizosphere microbial communities is still under debate (Lladó et al., 2017; Hartman and Tringe, 2019; Dove et al., 2021), a further step to investigate the relationship between host genotypes and rhizobacterial communities in woody plants is urgently required, which would provide a deeper insight into the potential utilization of soil microbes in trees breeding.

Poplar is very important for biofuel feedstock and timber production all over the world (Sannigrahi et al., 2010). However, the excellent poplar cultivar with high yield and quality wood is still in shortage in the practice of wood industry. Since the draft genome of black cottonwood (*Populus trichocarpa*) was published (Tuskan et al., 2006), it has been the model species in woody plants. And, genetic engineering based on molecular biology has markedly promoted the development of genetic-modified poplar breeding. However, as a dioecious tree species, hybridization between male and female parent lines still plays an important role in the practice of poplar selective breeding (Shen et al., 2021). For example, *Populus deltoides*, a rapid-growing poplar species with high timber production and tolerance to stress, is used as important parental lines in poplar cross-breeding. Because of the extensive distribution in mid-latitude countries and regions throughout the world, many natural variations of the species were observed and provided valuable genetic resources for modern poplar breeding (Fahrenkrog et al., 2017). Since some excellent germplasm resources of *P*. *deltoids* were introduced into China in the 1970s, the trees have formed many hybrid lineages with other numerous *Populus* species, and some of them displayed rapid growth and high yield even in some barren areas (e.g., arid and infertile sandy soils). To date, China has emerged as one of the countries with the largest plantations of *P. deltoides* in the world, contributing significantly to global wood production. The interaction between poplar and microbe has been paid more and more attention to in the sustainable development of poplar plantations. The examination of microbiomes related to poplar trees and the exploitation of core microbial communities have contributed much to poplar plantation silviculture. Currently, the profile of the poplar rhizosphere microbiome is becoming more and more clear (Schaefer et al., 2013; Wang et al., 2017). As dioecious tree species, their rhizobacterial communities were also shown to be significantly different between male and female poplar trees (Zhu et al., 2022), and their metabolic exudation of roots was also distinct (Xia et al., 2021). The temporal factors dominating the assembly of microorganisms associated with poplar have got extensive attention (Dove et al., 2021).

However, the limitations of previous studies are also obvious. For example, the observed trees were mostly young poplar, which limits the practical application of research achievements about beneficial microbes to mature poplar trees (Liu et al., 2021; Kristy et al., 2022). Obviously, the rhizosphere microbial communities are greatly affected by plant roots and appear to dramatically change with plant development and soil environmental fluctuations, which may finally disrupt the correct determination on the relationship between poplar and microbes. By contrast, mature poplar would provide a better model to investigate the interaction between trees and microbes (Shakya et al., 2013). Long-term interaction between trees and soils could develop a relatively stable ecosystem (Trivedi et al., 2020) and the impact of host genotype on soil microbiomes may emerge as tree ages (Dove et al., 2021). Additionally, the affinities among poplar genotypes in the previous studies were not clear, which may greatly influence the determination on the effect of poplar genotypes on microbial communities. Therefore, three mature poplar genotypes with well recorded parent lines were selected from a germplasm nursery of *P. deltoides* in this study. Their rhizobacterial communities were compared via 16S rDNA sequences, and the relationship between soil nutrients and rhizobacterial communities assembly was examined. Here, we tested two hypotheses, (1) the composition and structure of rhizobacterial communities would be different among poplar genotypes; and (2) the assembly of rhizobacterial communities may be determined by specific soil nutrients. The study would facilitate the integration of soil microbiomes with poplar breeding practice.

## Materials and methods

### Study site

The study site is located in the *Dashahe* national forestry farm of Shandong Province, 34°79’ (N) latitude and 116°08’ (E) longitude, with a warm temperate semi-humid continental monsoon climate and a mean annual temperature of 13.9°C. The frost-free period is approximately 206 d per year, and the annual average precipitation is approximately 737 mm. In terms of geographical location, the forestry farm is right on the Yellow River alluvial plain, and the soil texture mainly consists of sandy particles (more than 75%), which resulted in poor soil organic matter and nutrient conditions. Because the water- and fertilizer-holding capacity of the soil was extremely low, little irrigation and fertilization were applied in forest management. A germplasm nursery of *Populus deltoides* including 28 poplar genotypes was built in 2002, and the spacing between trees and rows was approximately 4 and 6 m, respectively. Twenty to thirty trees of each genotype were grown in a row, but all the genotypes regrettably had no replicates. Considering the influence of sex differences between male and female poplar trees on microbial communities (Xia et al., 2021; Zhu et al., 2022), the identical sex of poplar trees is required. In addition, to avoid soil heterogeneity, the poplar genotypes need to be neighboring as possible. Thus, only three male genotypes (H1, H2, and H3) were finally included in the study (Supplementary Figure 1A). According to the records of their parental lines, the three genotypic poplars are from four poplar parents (i.e., *P*. *deltoides* “Zhonghe 1,” “L23,” “T66,” and “I72”) and could be divided into three hybrid progeny groups (Supplementary Figure 1C): with the same female parent but a different male parent (SF, including H1 and H3); with the same male parent but a different female parent (SM, including H2 and H3); and without the same parent (NS, including H1 and H2). The understory vegetation of each genotype block mostly consisted of some common annual or perennial herbs, with no shrubs or other tree species.

### Poplar rhizosphere soil collection

Six sample trees were selected in each poplar genotype for rhizosphere soil collection. Briefly, five soil blocks (50 cm in length, 50 cm in width, and 20 cm in depth) were excavated at 0.5 m around the trunk based on the distribution of poplar fine roots (Supplementary Figure 1B; Zhu et al., 2018). After removing the surface herbaceous vegetation, poplar roots were collected from the soil block. Due to the absence of other woody plants in the plot, poplar roots can be easily distinguished from herbaceous roots based on their color and flexibility. The coarse roots of poplar in the soil block are helpful to assure that the fine roots were exactly from sample tree. However, only fine roots with a diameter of less than 2 mm were collected from coarse roots for this study. The fine roots were immediately placed in sterile bags and stored at 4°C for rhizosphere soil collection. Three quadrates (5 m × 5 m) were selected at random in each poplar genotype to obtain five bulk soil samples (Supplementary Figure 1B). Similarly, the bulk soils of three quadrates were mixed into a single sample. In the laboratory, a subset of fine roots from each sample tree was placed in a 50 mL centrifuge tube to which 30 mL PBS (phosphate buffer solution) was added and vortexed for 3 min. Following the removal of fine roots, rhizosphere soil was collected from tubes by centrifugation at 7,000 × *g* for 10 min (Li et al., 2014) and stored at −80°C for microbial communities analysis. Another subset of fine roots was collected for soil C, N and P analysis by brushing the fine root surface with a small brush. Thus, a total of 18 rhizosphere soil samples (6 tree samples × 3 genotypes) and 3 bulk soil samples were collected for this study.

### Soil carbon, nitrogen, and phosphorus analysis

The total carbon (TC) and total nitrogen (TN) contents of the soil samples were measured using an elemental analyzer (Vario MACRO cube, Elementar, Germany). The total phosphorus (TP) and available phosphorus (AP) contents in the soil samples were measured using an automatic chemical analyzer (SmartChem^®^ 200, AMS/Westco Scientific Instruments, Italy). The soil nitrate (NO_3_^–^) and ammonium (NH_4_^+^) contents were measured using an automatic flow analyzer (SEAL AutoAnalyzer AA3 HR, Germany). Finally, the soil stoichiometric characteristics were calculated.

### DNA extraction and amplification

Approximately 100 mg soil of each sample was taken from each sample for total soil microbial DNA extraction using the MAG-BIND Soil DNA Extraction Kit (Omega Bio-Tek, USA, Cat. # M5635-02). DNA quality was checked using an UV spectrophotometer (RS232G, Eppendorf, Germany). The V3-V4 region of bacterial 16S rDNA was amplified using the specific primers 338F (5′-ACTCCTACGGGAGGCAGCA-3′) and 806R (5′-GGACTACHVGGGTWTCTAAT-3′). The 250 bp paired-ends amplicons were sequenced using the Illumina platform (Illumina Inc., San Diego, California, USA).

### Bioinformatics

Sequence data were analyzed with QIIME 2 2019.4 (Bolyen et al., 2018). Briefly, raw data were demultiplexed using the demux plugin, followed by primers removal with the cutadapt plugin (Martin, 2011). Sequences were delineated into amplicon sequence variants (ASVs) after quality filtration, mergence, and chimera sequence removal using the DADA2 algorithm (Callahan et al., 2016). According to the Silva database, the representative sequences were assigned a taxonomic classification using the classify-sklearn na1̈ve Bayes taxonomy classifier, and then the host contaminants (e.g., chloroplast or mitochondrial sequence) were removed. Finally, a total of 11,733 ASVs were obtained. Using the QIIME feature-table rarefy function, these ASVs were randomly selected according to 95% of the minimum sample sequence size to obtain the rarefied ASV table (Kemp and Aller, 2004). And then, the relative abundance of ASV, which is the amount of ASV in a sample as a percentage of the total abundance of that sample, was calculated for subsequent analysis. The raw data were submitted to the NCBI Sequence Read Archive (SRA) database (PRJNA881800).

### Statistical analysis

All statistical analyses and plots in this study were generated in R 4.0.5 (R Core Team, 2020). One-way analysis of variance (ANOVA) and least significant difference (LSD) multiple comparisons were used to test the differences in soil nutrition content, α diversity (Chao1, ACE, Shannon, and PD index), NTI of bacterial communities among groups (α = 0.05). Linear discriminant analysis^1^ was used to identify biomarkers at different taxonomic levels. Correlation analysis was performed using the ‘‘psych’’ package (V.2.0.9)^2^ and visualized using the ‘‘corrplot’’ package (V.0.84).^3^ The ‘‘TITAN2^4^ “package was used for threshold indicator taxa analysis to determine the response of the bacterial communities to environmental factors. This method determines the response threshold of the soil bacterial community based on the change points of each taxon along the environmental gradient and the synchronization of the change points (Gao et al., 2020). Gephi software (V.0.9.2)^5^ was used to visualize and analyze the microbial co-occurrence network (Bastian et al., 2009). Taxa with 0 abundance were excluded from the data for each group, and only significant correlations were included in the network (*r* > 0.8, *P* < 0.01). Nodes with higher within-module connectivity (Zi) and among-module connectivity (Pi) were defined as hub nodes (Shi et al., 2016). Phylogenetic trees were constructed using FastTree2 software (Price et al., 2010), and the nearest taxon index (NTI) was calculated using the “picante” package (V.1.8.2) (Kembel et al., 2010). The NTI was used to assess phylogenetic processes; NTI > 0 indicates phylogenetic clustering, and NTI < 0 indicates phylogenetic overdispersion (Kembel, 2009). The assembly processes of the microbial community were evaluated using a null model (Stegen et al., 2012). The standard deviation of the observed β-mean-nearest taxon distance (βMNTD) from the mean of the null distribution is defined as the β-nearest taxon index (βNTI). | βNTI| > 2 indicates the dominance of deterministic processes, including homogeneous selection (βNTI < −2) and variable selection (βNTI > 2), and | βNTI| < 2 indicates the dominance of stochastic processes. The source code is available at https://github.com/yankun212/Frontiers_PGR.git.

## Results

### Comparisons of carbon, nitrogen, and phosphorus contents in rhizosphere soils of poplar genotypes

The NO_3_^–^-N content in the rhizosphere soil of H2 was more than those of other soils, while the NH_4_^+^-N/NO_3_^–^-N ratio was less (Table 1). Specifically, the NO_3_^–^-N content of H1 and H2 rhizosphere soils differed significantly (*P* < 0.05). Similarly, the content of NO_3_^–^-N and NH_4_^+^-N/NO_3_^–^-N ratio of H2 and H3 showed significant differences (*P* < 0.05). However, the nutrient content and stoichiometric characteristics of rhizosphere soils did not differ significantly between H1 and H3. Therefore, the soil available nitrogen contents appeared closely relative to poplar genotypes.

**TABLE 1:** The soil carbon, nitrogen, phosphorus content, and stoichiometric characteristic in rhizosphere and bulk soils of poplar genotypes (mean ± SE).

	TC (mg⋅g^–^^1^)	TOC (mg⋅g^–^^1^)	TN (mg⋅g^–^^1^)	TP (mg⋅g^–^^1^)	AP (μg⋅g^–^^1^)	NO_3_^–^-N (μg⋅g^–^^1^)	NH_4_^+^-N (μg⋅g^–^^1^)	NH_4_^+^: NO_3_^–^	TC:TN	TC:TP	TN:TP	TC: TN:TP
H1	13.33 ± 0.86a	6.55 ± 0.57a	0.78 ± 0.11a	0.46 ± 0.01a	2.23 ± 0.45a	23.06 ± 2.93b	14.18 ± 1.81ab	0.65 ± 0.10a	18.39 ± 2.11a	29.01 ± 1.62a	1.70 ± 0.22a	40.26 ± 4.77a
H2	13.14 ± 0.38a	6.41 ± 0.52a	0.74 ± 0.04a	0.44 ± 0.01a	0.96 ± 0.38a	27.22 ± 1.82a	11.94 ± 1.54b	0.46 ± 0.08b	17.99 ± 0.64a	29.71 ± 0.56a	1.66 ± 0.05a	41.00 ± 2.73a
H3	13.03 ± 1.17a	6.46 ± 1.10a	0.72 ± 0.12a	0.47 ± 0.01a	1.50 ± 0.53a	24.86 ± 0.62b	15.70 ± 1.09ab	0.72 ± 0.11a	19.40 ± 1.64a	27.58 ± 2.61a	1.53 ± 0.27a	40.76 ± 2.84a
BS	13.12 ± 0.46a	7.34 ± 0.51a	0.78 ± 0.11a	0.46 ± 0.01a	2.66 ± 1.12a	22.01 ± 2.35b	18.50 ± 1.96a	0.86 ± 0.13a	17.36 ± 1.7a	28.30 ± 1.25a	1.68 ± 0.25a	37.31 ± 3.21a

Different letters in the same column mean significant difference among the different soil compartments at 0.05 level. Total carbon (TC), total organic carbon (TOC), total nitrogen (TN), total phosphorus (TP), available phosphorus (AP), and the ratio of NH_4_^+^-N to NO_3_^–^-N (NH_4_^+^-N: NO_3_^–^-N).

### Comparisons of rhizobacterial community diversity among poplar genotypes

**FIGURE 1:**
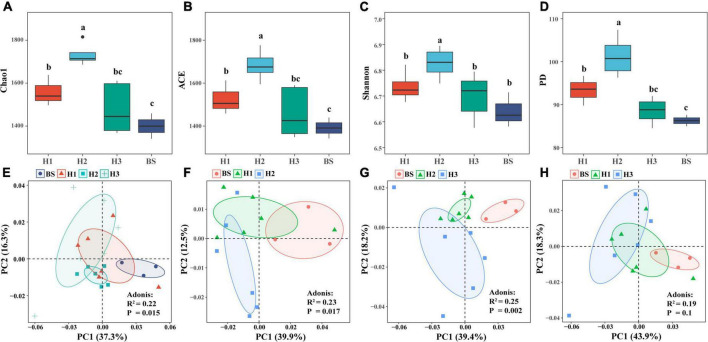
α-diversity **(A–D)** and β-diversity **(E–H)** of bacterial community in rhizosphere and bulk soils of poplar genotypes.

Analysis of rhizobacterial community diversity revealed that the richness index (Chao1, ACE) and phylogenetic index (PD) of H1 and H2 differed significantly (*P* < 0.05), as did those of H2 and H3. However, there were no significant differences of diversity indices between H1 and H3 (Figures 1A–D). These findings indicated that the structures of rhizobacterial communities differed among poplar genotypes. Furthermore, principal component analysis (PCA) showed significant differences in the rhizobacterial community between H1 and H2 (*R*^2^ = 0.23, *P* = 0.017), as well as between H2 and H3 (*R*^2^ = 0.25, *P* = 0.002), but not between H1 and H3 (*R*^2^ = 0.19, *P* = 0.1) (Figures 1E–H). The three tests of Adonis, ANOSIM, and MRPP revealed identical differences among the three poplar genotypes (Supplementary Table 1). The results indicated that the structure of rhizobacterial communities was significantly related to poplar genotypes.

### Differences in the composition of rhizobacterial communities among poplar genotypes

**FIGURE 2:**
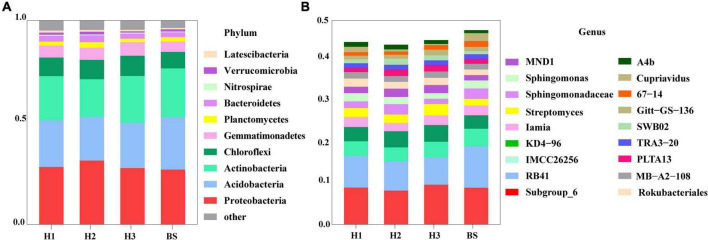
The composition of bacterial community in rhizosphere and bulk soils at the level of phylum **(A)** and genus **(B)**.

**FIGURE 3:**
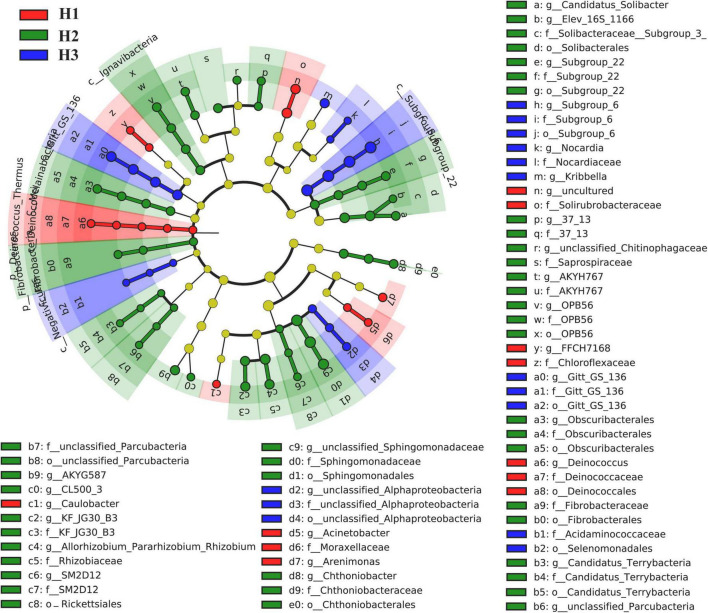
Linear discriminant effect size analysis (LEfSe) on the rhizobacteria biomarkers of poplar genotypes.

**FIGURE 4:**
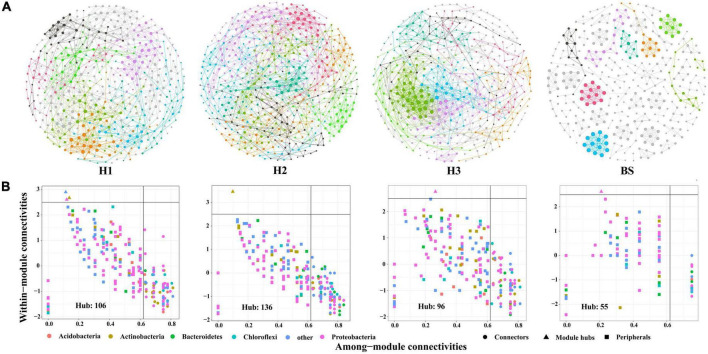
The co-occurrence networks **(A)** and hub nodes **(B)** of bacteria in rhizosphere and bulk soils. Different colors in the network represent different modules.

A total of 36 phyla and 666 genera of bacteria were annotated in all the sample soils, with 10 phyla showing a relative abundance of over 1% (Figure 2). Proteobacteria and Planctomycetes had significantly different relative abundances between H1 and H2, as well as between H2 and H3 (*P* < 0.05). However, the relative abundance of the above two bacterial phyla did not differ between H1 and H3 (*P* > 0.05). The F-values of nine abundant phyla in the SF group (H1 and H3 genotype) were smaller than those of the other two groups (SM and NS) (Supplementary Table 2). The VENN diagram showed that the number of rhizobacteria ASVs shared by H1 and H3 (741 + 1,705) was greater (Supplementary Figure 2). Linear discriminant effect size analysis indicated that there were 11 and 14 specific biomarkers in the H1 and H3 genotypes, respectively, while it was up to 42 biomarkers in the H2 genotype (Figure 3). So, H1 and H3 had a similar composition and structure of rhizobacterial communities and were different from the H2 genotype. Additionally, co-occurrence network analysis showed that H1 and H3 had similar average network connectivity and modularity (Figure 4). However, H2 had more network nodes, edges, and higher average degrees (Supplementary Table 3). The above results suggested that the composition and structure of rhizobacterial communities in poplar genotypes were different.

### Correlation between rhizobacterial community and soil nutrients

**FIGURE 5:**
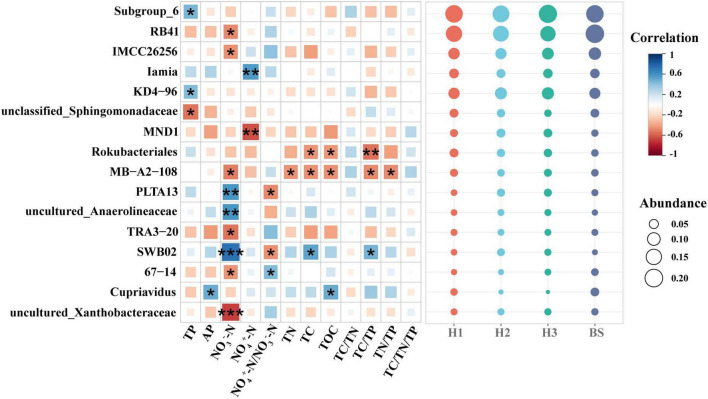
The correlation matrix between soil nutrient properties and abundant bacteria genera (the relative abundance over 1%) in rhizosphere and bulk soils of poplar. ***, ** and * show significant correlation at the 0.001, 0.01 and 0.05 level, respectively.

**FIGURE 6:**
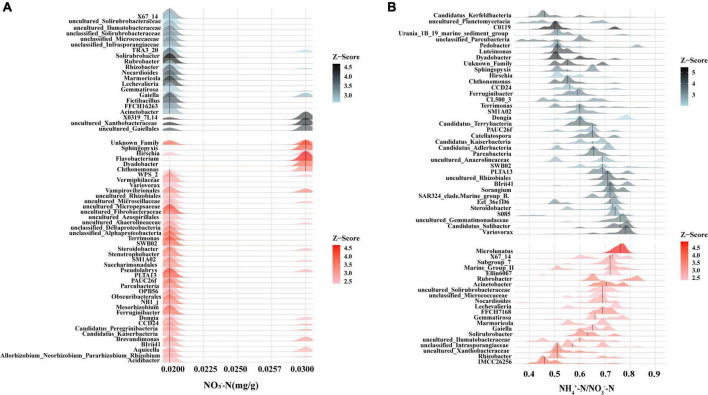
Threshold indicator taxa analysis (TITAN) on the response of bacteria genus to NO_3_^–^-N content **(A)** and the ratio of NH_4_^+^-N/NO_3_^–^-N **(B)**. Red color represents positive response (i.e., abundance increases), black color represents negative response (i.e., abundance decrease).

Among the rhizobacteria genera with a relative abundance over 1%, 16 genera demonstrated a significant correlation with soil nutrients, and the majority of genera (up to nine genera) demonstrated a significant correlation with soil NO_3_^–^-N content (Figure 5). Meanwhile, threshold indicator taxa analysis revealed that 63 genera were responsive to NO_3_^–^-N changes and 58 genera were responsive to NH_4_^+^-N/NO_3_^–^-N changes (Figure 6). These findings indicate that the content of NO_3_^–^-N and the ratio of NH_4_^+^-N/NO_3_^–^-N have a significant impact on the composition of the poplar rhizobacterial community.

**FIGURE 7:**
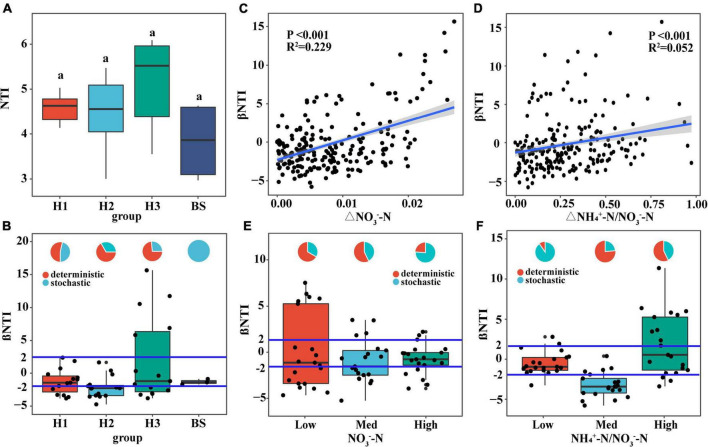
The relationship between the available nitrogen and the assembly of soil bacterial communities. Distribution of NTI value **(A)** and βNTI value **(B,E,F)** of soil bacteria community, and the relationship of βNTI value **(C,D)** with the difference of NO_3_^–^-N content and NH_4_^+^-N/NO_3_^–^-N. The small graphs in panels **(B,E,F)** represent the proportions of stochastic and deterministic processes in different groups of bacterial communities.

NTI of rhizobacterial communities did not show significant differences among poplar genotypes (Figure 7A), indicating phylogenetic clustering of rhizobacterial taxa in the poplar genotype (NTI > 0). Furthermore, the rhizobacterial assembly was dominated by deterministic processes, however, the bacterial assembly of bulk soil was dominated by stochastic processes (Figure 7B). Pairwise comparisons of βNTI had a positive relationship with the difference of soil NO_3_^–^-N content (*R*^2^ = 0.229; *P* < 0.001) and NH_4_^+^-N/NO_3_^–^-N ratio (*R*^2^ = 0.052; *P* < 0.001) (i.e., the Euclidean dissimilarity) (Figures 7C,D), indicating that changes in soil nutrient content drive the transformation of bacterial assembly patterns. Furthermore, it is found that stochastic processes decreased with increasing NO_3_^–^-N content and fluctuated with increasing NH_4_^+^-N/NO_3_^–^-N ratio (Figures 7E,F). NO_3_^–^-N may be one of the dominant edaphic factors influencing bacterial community assembly in poplar rhizosphere soils.

## Discussion

### The differences of rhizobacterial community among progenies

Evidence suggests that differences of plant rhizosphere microbial communities are associated with the rhizosphere environment (Naylor et al., 2017; Wang et al., 2018). NO_3_^–^-N is an important form of available nitrogen which is directly utilized by the majority of plant species (Von Wirìn et al., 1997). The present study showed that the NO_3_^–^-N content in the rhizosphere soil was significantly different among the poplar genotypes (Table 1), implying the utilization or transformation of NO_3_^–^-N in the rhizosphere soil is related to poplar genotypes. Further, 9 of 16 bacterial genera with a relative abundance of over 1% had a significant correlation with NO_3_^–^-N (Figure 5), implying that poplar trees recruited a large number of microorganisms in the rhizosphere for nitrogen cycling. Studies have shown that plants usually provide various substrates for rhizosphere microorganisms via root exudation (Bakker et al., 2018; Williams and de Vries, 2020) to recruit some beneficial microorganisms (e.g., *Bacillus subtilis* GB03, *Bacillus subtilis* FB17) in the rhizosphere soil (Bulgarelli et al., 2012; Hashem et al., 2019; Matthews et al., 2019), which was helpful to improve soil nutrient availability and the adaptation of plants to specific environments (Berendsen et al., 2012; Trivedi et al., 2020). For example, the profiles of root metabolism revealed that the influences of poplar root phenolic metabolomes on rhizobacterial communities were dependent on poplar sex and soil properties (Xia et al., 2021, 2022). Meanwhile, soil nutrient availability also affect rhizosphere microbial communities (Delgado-Baquerizo et al., 2017). According to the growth rate hypothesis, the carbon and nitrogen substrates from roots simultaneously affect the soil ecological process (i.e., rhizosphere priming effect) (Lu et al., 2019) and demonstrate significant regulation of soil microorganisms (Nyawade et al., 2019). Additionally, other soil elements (e.g., phosphorus) also play an important role in microbial colonization and plant growth (Graham, 2002; Peñuelas and Sardans, 2009). So, the rhizobacterial communities are dependent on soil nutrient conditions.

Meanwhile, rhizosphere microbial communities are closely related to plant genotypes (Bokulich et al., 2014; Rochefort et al., 2019). For example, the diversity and co-occurrence networks of rhizosphere microbial communities were shown differently among genotypes of Arabidopsis (*Arabidopsis thaliana*) and blueberry (*Vaccinium ashei*), respectively (Lundberg et al., 2012; Jiang et al., 2017). Mycorrhizal infections were significantly increased by secreted enzyme activities (e.g., β-xylosidase, cellobiohydrolase, β-glucosidase, acid phosphatase, and laccase) in mycorrhizal roots of different poplar genotypes (Courty et al., 2010). Based on the available literature, the differences in rhizosphere bacterial communities among plant cultivars are often observed (Zhu et al., 2012; Bokulich et al., 2014; Li et al., 2014). In the study, we also found that the rhizobacterial compositions were different among the three genotypes of *P. deltoides* (Figure 2E). This difference may be attributable to the various root exudates of different genotypic plants (Matthews et al., 2019). However, there are still few studies that could provide enough evidence about the genetic effects of parental lines on rhizosphere microbiomes of plant genotypes, except that ectomycorrhizal fungal communities of hybrid progenies were observed to be different from parents in *Populus* (Lamit et al., 2021). In this study, three poplar genotypes can be classified into three hybrid progeny groups based on their parental lines (Supplementary Figure 1). Thus, we attempted to clarify the potential effect of hybridization on the assembly of poplar rhizobacterial communities. We observed significant differences in rhizobacterial community diversity both in NS and SM groups (Figures 1A,B,D,F). However, the similar composition and diversity of the poplar rhizobacterial community were shown in the SF group (Figure 1H). Further analysis revealed that the number of co-shared ASVs in the SF group was greater than those in the SM and NS groups, whereas it was comparable between the NS and SM groups (Supplementary Figure 2). Meanwhile, the least biomarkers and the highest degree of similarity were observed in the H1 and H3 (SF group) (Figure 3), as well as a similar co-occurrence network of rhizobacterial communities (Figure 4). All these findings suggest that the rhizobacterial community composition of poplar genotypes may be related to the genetic effects of their parental lines. Regrettably, the results of this study are only observational and not from a formally designed field experiment. However, we believe that common garden experiments are helpful to examine the differences in rhizosphere microbial communities between progenies and parents in the future, which would provide more accurate and reliable information about the genetic effects of poplar on rhizobacterial communities.

### Assembly process of rhizobacterial communities and its dominant factors

Quantifying the assembly process of microbial communities is important in microbial ecology research (Zhou and Ning, 2017). Community assembly of environmental microorganisms can be divided into stochastic and deterministic process. The stochastic process is random diffusion, while the deterministic process is environment selection, which explains the different responses of microbes to specific niches. Meanwhile, stochastic and deterministic process may accompany the entire life history of a host, but the balance between them is often influenced by environmental factors (Jiao et al., 2020). In harsh environments (such as limited nutrient resource), natural selection pressure limits the diversity and abundance of bacterial communities (Zhou and Ning, 2017), and the assembly of rhizosphere bacteria was dominated by a deterministic process (Dove et al., 2021). Our results indicated that the assembly of bacterial communities were influenced by a stochastic process in bulk soil but a deterministic process in rhizosphere soil (Figure 7). The difference may be attributable to the distinct soil properties. Compared to bulk soils, plant rhizosphere is a very complex micro-environment affected by many factors (e.g., root morphology, nutrient utilization preference of plant, and root exudation) (Gottel et al., 2011; Dove et al., 2021; Herms et al., 2022). During plant growth and development, plant roots can selectively recruit microorganisms colonizing the rhizosphere or rhizoplane via dead residues and root exudation (Hartmann et al., 2009), resulting in significant differences in microbial communities between bulk soil and rhizosphere soil (Gregory, 2006; Uroz et al., 2010). So, the richness of the bacterial communities in rhizosphere soil was significantly greater than that in bulk soil, and the assembly process of bacterial communities may be different between the two soil environments.

In tandem with the root-soil interaction process, the assembly of the rhizobacterial community is constantly in a state of flux. Study have also shown that the functional requirements (e.g., soil nutrients) of the host plant play an important role in the assembly of rhizobacterial communities (Ren et al., 2020). Soil available nutrients and their stoichiometry can drive the changes in soil microbial communities (Delgado-Baquerizo et al., 2017). Nitrogen is very important for rapid-growth poplar trees. In this study, the NO_3_^–^-N contents in the rhizosphere soils of different poplar genotypes were found to be significantly different (Table 1), which may greatly affect the assembly of rhizobacteria communities. Although there was no significant difference in rhizobacterial NTI among poplar genotypes (Figure 7A), βNTI was significantly correlated positively with the differences of soil NO_3_^–^-N content and the NH_4_^+^-N/NO_3_^–^-N ratio (Figures 7B,C). Thus, when available nitrogen in the rhizosphere is deficient, microbial growth is limited so that both positive and negative priming effects occur in the rhizosphere (Dijkstra et al., 2013). At this time, some rhizobacteria are recruited through deterministic heterogeneous selection, such as *Nitrosospira* (Zhang et al., 2019), *Noviherbaspirillum* (Ishii et al., 2017), and *Mesorhizobium* (Siddiqi et al., 2019), which accelerates soil mineralization to meet the plant’s demand for available nutrients (Blagodatskaya et al., 2007). When the available nitrogen in the rhizosphere is abundant, plants secrete large amounts of carbohydrates into rhizosphere soils. Rhizosphere microbes would use these root exudates as nitrogen and carbon substrates (Kuzyakov and Cheng, 2004; Dijkstra et al., 2013). Consequently, the rhizobacterial community tended to be randomly assembled across nitrogen content gradients (Figures 7E,F). All the preceding findings suggest that available nitrogen may be a dominant edaphic factor influencing the assembly of rhizobacterial communities.

## Conclusion

This study revealed that the available nitrogen content in rhizosphere soil varied among the poplar genotypes. Moreover, the composition and structure of the rhizobacterial community were closely related to the poplar genotypes. Soil available nitrogen may play a significant role in determining the assembly of the rhizosphere bacterial community. The study could aid in the development of plant-microbe symbiotic breeding in the future.

## Data availability statement

The datasets presented in this study can be found in online repositories. The names of the repository/repositories and accession number(s) can be found below: https://www.ncbi.nlm.nih.gov/, PRJNA881800.

## Author contributions

QZ, KY, NW, SM, DL, and ZY conducted the field work, data collection, and statistical analysis. QZ and KY conducted visualization and writing. XS, YW, YD, and CD conducted methodology design, data curation, and manuscript reviewing and writing. YW and CD executed the figure drawing and manuscript writing. All authors read and approved the final manuscript.

## Footnotes

1
http://huttenhower.sph.harvard.edu/galaxy/

2
https://CRAN.R-project.org/package=psych

3
https://github.com/taiyun/corrplot

4
https://CRAN.R-project.org/package=TITAN2

5
https://gephi.org
