# Supplementary material for: The structure and assembly of rhizobacterial communities are influenced by poplar genotype
Source: Front Microbiol. 2022 Nov 29;13:1052567. doi: 10.3389/fmicb.2022.1052567 (PMC9746989; doi:10.3389/fmicb.2022.1052567)
Supplement: Supplementary file 1 [file Data_Sheet_1.docx]

Supplementary Materials

# Supplementary Figures and Tables

## Supplementary Figures

**Figure S1: (A) 28 genotypes planted nursery. (B) Schematic diagram of rhizosphere and bulk soils sampling. (C)The examined poplar genotypes and progeny groups based their parent lines. (D) Photos from the field.**

**
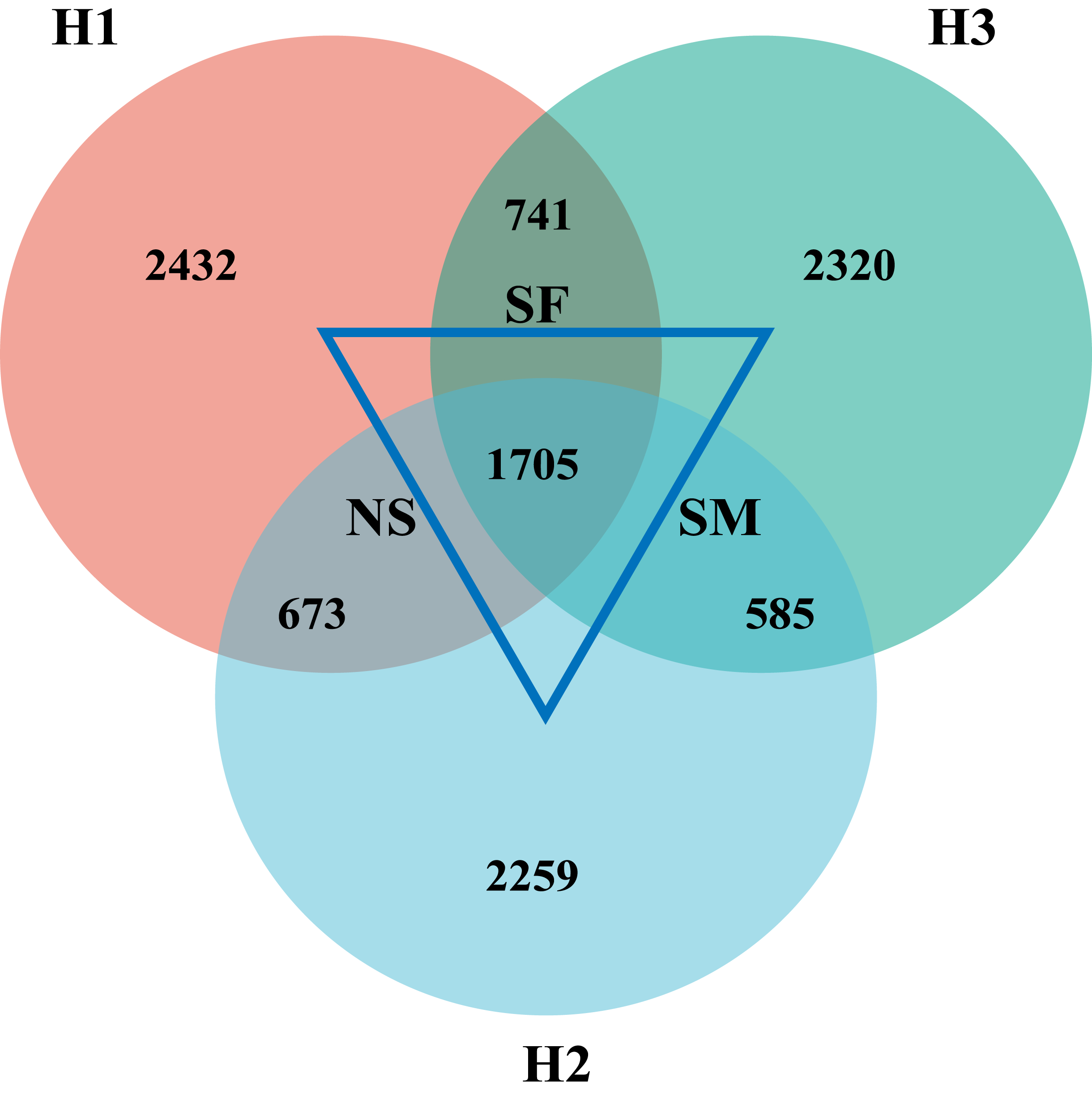
**

**Figure S2:The Venn diagram of bacteria ASVs in rhizosphere and bulk soils.**

## Supplementary Tables

**Table S1 Adonis, ANOSIM, MRPP analysis on the β-diversity of rhizobacterial community among three poplar genotypes.**

|  | Adonis | | ANOSIM | | MRPP | |
| --- | --- | --- | --- | --- | --- | --- |
|  | R^2^ | Pr (> F) | R | P | A | P |
| H1vs H2 vs H3 | 0.217 | 0.015* | 0.213 | 0.009** | 0.070 | 0.006** |
| H1vs H2(NS) | 0.228 | 0.017** | 0.275 | 0.020* | 0.074 | 0.021* |
| H1vs H3(SF) | 0.189 | 0.1 | 0.083 | 0.212 | 0.056 | 0.059 |
| H2vs H3(SM) | 0.245 | 0.002** | 0.348 | 0.002** | 0.11 | 0.001*** |

*** P <0.05, ** P < 0.01, and *** P < 0.001.**

**Table S2 The variance analysis on rhizosphere bacterial community among progeny groups at phylum-level**

|  | H1 vs. H2 (NS) | |  | H1 vs. H3 (SF) | |  | H2 vs. H3 (SM) | |
| --- | --- | --- | --- | --- | --- | --- | --- | --- |
| Phylum | F statistic | P value |  | F statistic | P value |  | F statistic | P value |
| Proteobacteria | **5.641** | **0.039*** |  | **0.047** | 0.834 |  | 3.054 | 0.111 |
| Actinobacteria | 3.564 | 0.088 |  | **0.286** | 0.605 |  | 4.525 | 0.059 |
| Acidobacteria | 1.008 | 0.339 |  | **0.157** | 0.7 |  | 0.208 | 0.658 |
| Chloroflexi | 0.147 | 0.71 |  | 0.799 | 0.392 |  | 0.507 | 0.493 |
| Gemmatimonadetes | 0.031 | 0.864 |  | 4.368 | 0.063 |  | 4.887 | 0.052 |
| Bacteroidetes | 1.426 | 0.26 |  | **0.351** | 0.567 |  | 2.161 | 0.172 |
| Rokubacteria | 1.088 | 0.321 |  | 0.331 | 0.578 |  | 0.076 | 0.788 |
| Planctomycetes | 4.693 | 0.056 |  | **0.231** | 0.641 |  | **7.022** | **0.024*** |
| Patescibacteria | 3.762 | 0.081 |  | **0.088** | 0.772 |  | 0.494 | 0.498 |
| Latescibacteria | 0.905 | 0.364 |  | 3.044 | 0.112 |  | 1.538 | 0.243 |
| Firmicutes | 2.41 | 0.152 |  | **0.006** | 0.941 |  | 0.981 | 0.345 |
| Verrucomicrobia | 1.321 | 0.277 |  | 2.095 | 0.178 |  | 4.867 | 0.052 |
| Nitrospirae | 0.231 | 0.641 |  | **0.024** | 0.88 |  | 0.181 | 0.679 |
| Armatimonadetes | 0.029 | 0.868 |  | 0.087 | 0.774 |  | 0.138 | 0.718 |
| Elusimicrobia | 0.823 | 0.386 |  | 2.771 | 0.127 |  | 0.235 | 0.638 |
| Dependentiae | 0.41 | 0.537 |  | **0.139** | 0.717 |  | 0.765 | 0.402 |

*** P <0.05, ** P < 0.01, and *** P < 0.001.**

**Table S3 The comparison on topological properties of co-occurrence network of rhizobacteria communities among three poplar genotypes**

|  | Node | Edge | Average degree | Modularity | Average clustering coefficient | Average path length | Hub node |
| --- | --- | --- | --- | --- | --- | --- | --- |
| H1 | 469 | 1343 | 5.737 | 0.78 | 0.456 | 6.276 | 106 |
| H2 | 504 | 1491 | 5.992 | 0.771 | 0.414 | 6.069 | 136 |
| H3 | 420 | 1377 | 5.588 | 0.68 | 0.431 | 6.253 | 96 |
| BS | 353 | 777 | 4.524 | 0.96 | 0.85 | 1.966 | 55 |
